# Small Molecule Tools to Study Cellular Target Engagement for the Intracellular Allosteric Binding Site of GPCRs

**DOI:** 10.1002/chem.202202565

**Published:** 2022-11-08

**Authors:** Max E. Huber, Lara Toy, Maximilian F. Schmidt, Dorothee Weikert, Matthias Schiedel

**Affiliations:** ^1^ Department of Chemistry and Pharmacy Medicinal Chemistry Friedrich-Alexander-Universität Erlangen-Nürnberg (FAU) Nikolaus-Fiebiger-Straße 10 91058 Erlangen Germany

**Keywords:** chemical biology, drug discovery, fluorescent probes, GPCRs, receptors

## Abstract

A conserved intracellular allosteric binding site (IABS) has recently been identified at several G protein‐coupled receptors (GPCRs). Ligands targeting the IABS, so‐called intracellular allosteric antagonists, are highly promising compounds for pharmaceutical intervention and currently evaluated in several clinical trials. Beside co‐crystal structures that laid the foundation for the structure‐based development of intracellular allosteric GPCR antagonists, small molecule tools that enable an unambiguous identification and characterization of intracellular allosteric GPCR ligands are of utmost importance for drug discovery campaigns in this field. Herein, we discuss recent approaches that leverage cellular target engagement studies for the IABS and thus play a critical role in the evaluation of IABS‐targeted ligands as potential therapeutic agents.

## Graphical abstract

**A druggable intracellular allosteric binding site** (IABS) has recently been identified at several GPCRs. Ligands targeting the IABS are promising drugs and currently evaluated in several clinical trials. Herein, we review small molecule tools that enable an unambiguous identification and characterization of cellular target engagement for intracellular allosteric GPCR ligands.

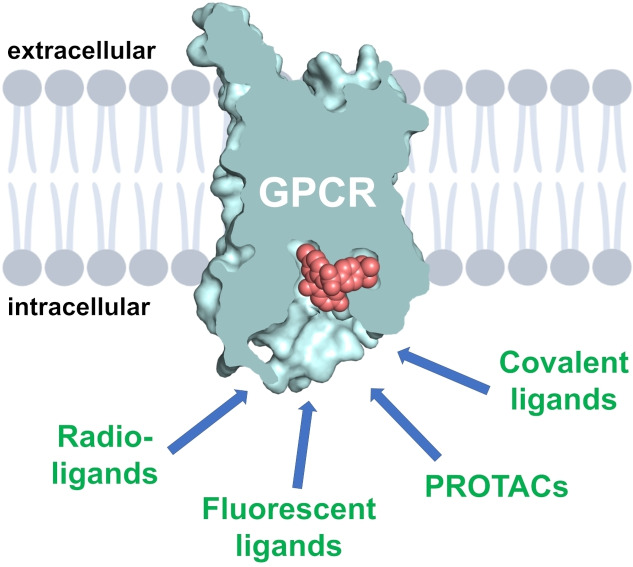

## Introduction

**Figure 1:**
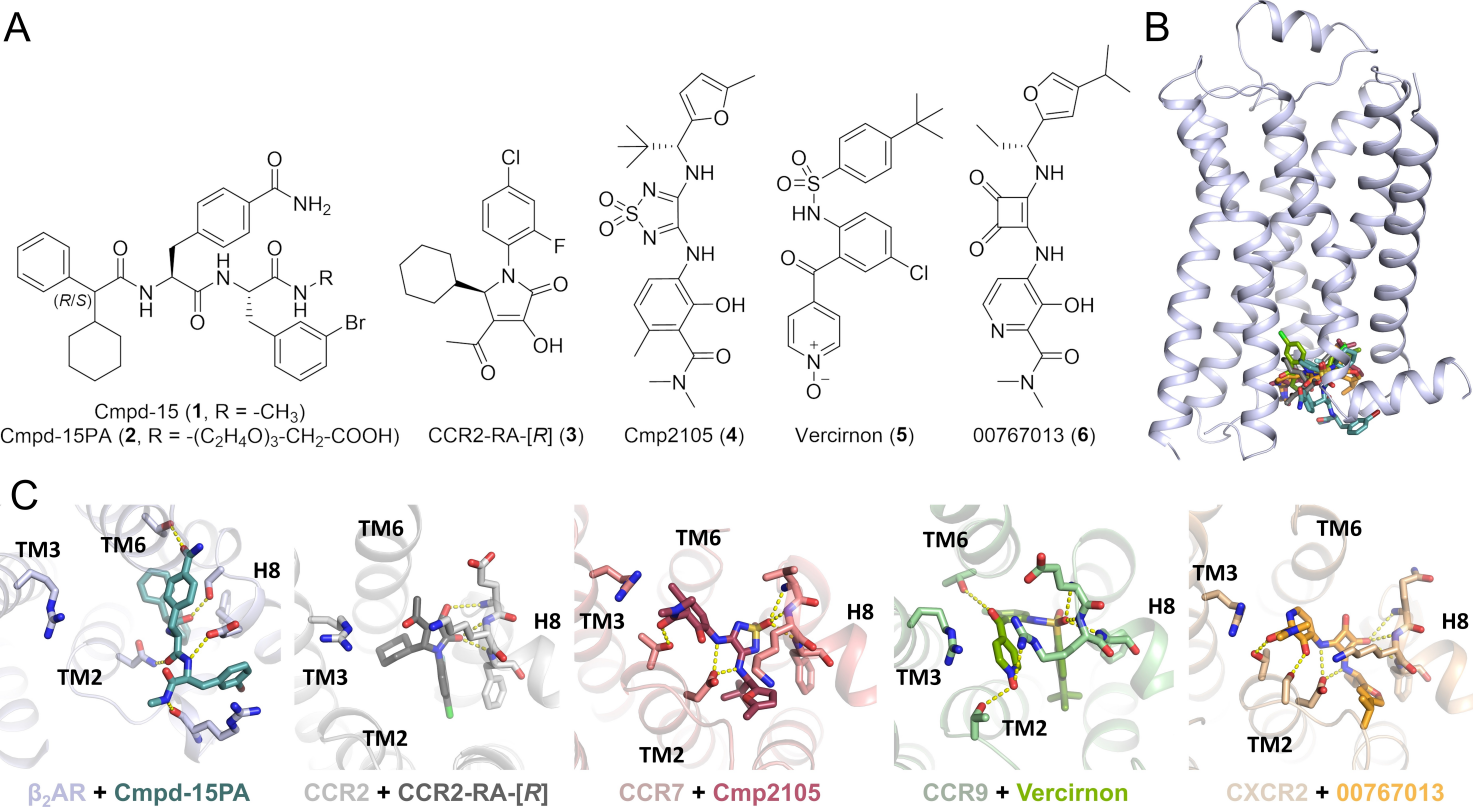
Small molecule GPCR antagonists targeting a highly conserved intracellular allosteric binding site (IABS). A) Chemical structures of allosteric antagonists **1–6** binding to the IABS of their targeted GPCR.[^2^, ^3^, ^4^, ^5^, ^6^] B) Location of the highly conserved intracellular allosteric site. The representative crystal structure of β_2_AR‐Cmpd‐15PA complex (PDB: 5X7D) was selected as a template, and the other crystal structures of GPCR−intracellular allosteric antagonist complexes (CCR2‐CCR2‐RA‐[*R*] (PDB: 5T1A), CCR7‐Cmp2105 (PDB: 6QZH), CCR9‐vercirnon (PDB: 5LWE), and CXCR2‐00767013 (PDB: 6LFL) are superimposed onto the β_2_AR−Cmpd‐15PA complex. The β_2_AR is colored in blue, and the intracellular allosteric antagonists are shown in stick representation (Cmpd‐15PA ((*R*)‐epimer (teal), the PEG carboxylic acid is not resolved in the structure), CCR2‐RA‐[*R*] (grey), Cmp2105 (red), vercirnon (green), 00767013 (orange)). In the interest of clarity, co‐crystallized orthosteric ligands are not shown. C) Comparison of the binding modes of intracellular allosteric antagonists. H‐bond interactions are shown as dashed lines (yellow). The PEG‐carboxylic acid of Cmpd‐15PA is not resolved in the structure.

G protein‐coupled receptors (GPCRs) are one of the most relevant protein families in drug discovery, as they are targeted by approximately one third of all available medications.^[1]^ The vast majority of known GPCR ligands binds to an orthosteric site located within the helical bundle, facing the extracellular side of the receptor. Apart from the orthosteric site, a highly conserved intracellular allosteric binding site (IABS) that enables the binding of small molecule antagonists (**1**–**6**) has recently been identified by means of X‐ray co‐crystallography for the beta‐2 adrenergic receptor (β_2_AR)^[2]^ and the chemokine receptors CCR2,^[3]^ CCR7,^[4]^ CCR9,^[5]^ and CXCR2 (Figure 1).^[6]^ In addition, a druggable IABS has been suggested for several other class A GPCRs.^[7]^ A very recent computational study by Hedderich et al. even supports the existence of a druggable IABS beyond class A GPCRs.^[8]^

**Figure 2:**
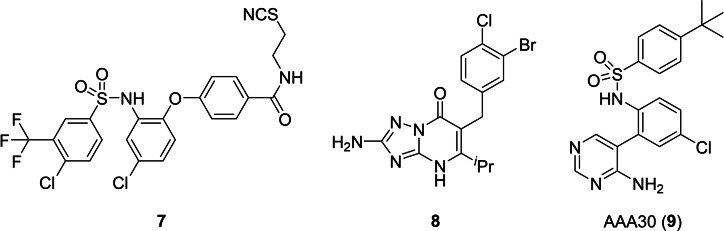
Representative chemical structures of intracellular GPCR antagonists that have been developed in a structure‐based approach, using the information provided by the respective co‐crystal structures.[^3^, ^5^] Compound **7** is a covalent CCR2 antagonist, **8** a dual CCR2/CCR5‐antagonist, and AAA30 (**9**) a selective CCR9‐targeted antagonist.

Ligands targeting the IABS, so‐called intracellular allosteric antagonists, inhibit GPCR‐mediated signaling via a new dual mechanism. This new mode of specific GPCR modulation is characterized by a stabilization of the inactive receptor conformation and a steric blockage of intracellular transducer (G protein and/or β‐arrestin) binding.[^2^, ^3^, ^4^, ^5^] Thus, targeting the IABS opens new opportunities to modulate receptor activity, receptor selectivity, as well as functional selectivity. In pre‐clinical and clinical studies, intracellular allosteric antagonists, such as the CCR9‐targeted vercirnon showed very promising effects, especially for the treatment of inflammatory diseases. Vercirnon even progressed to phase III clinical trials for the treatment of Crohn's disease, however, ultimately failed at this late stage of clinical development due to limited therapeutic efficacy,^[9]^ thereby exemplifying the high therapeutic potential but also the current limitations of intracellular GPCR antagonists. The vast majority of the intracellular allosteric GPCR antagonists, which are comprehensively reviewed in the overview articles of Billen et al.^[10]^ and Ortiz Zacarías et al.,^[7]^ have already been identified and chemically optimized in the absence of any detailed structural information on intracellular GPCR antagonism. The recent disclosure of the co‐crystal structures of β_2_AR,^[2]^ CCR2,^[3]^ CCR7,^[4]^ CCR9,^[5]^ CXCR2^[6]^ in complex with small molecule intracellular antagonists laid the foundation for the structure‐based development of further intracellular GPCR antagonists, such as the covalent CCR2 antagonist **7**,^[11]^ the dual CCR2/CCR5‐antagonist **8**,^[12]^ or the CCR9‐targeted AAA30 (**9**, Figure 2).^[13]^

Besides structural insights on receptor‐ligand interactions, molecular tools that enable an unambiguous identification and characterization of intracellular GPCR ligands are highly important for drug discovery campaigns in this field. This is supported by the fact that the structure‐based development of **7**–**9** was guided by binding assay data generated with hand‐tailored molecular tools targeting the IABS of the respective receptor.[^11^, ^12^, ^13^]

In general, a critical step in preclinical drug discovery is the assessment of the interactions between a drug and its protein target in a cellular environment. This step, also referred to as cellular target engagement, is highly relevant for successfully delivering compounds with the desired biological and ultimately clinical effects, as the on‐target activity of a drug can be changed significantly when transitioning from a biochemical in vitro setup to a live cell environment. In a cellular environment, the activity of a drug can be affected by various factors, such as low cell permeability, compound efflux, and off‐target binding. As intracellular allosteric GPCR antagonists need to pass the cell membrane to reach their binding site, molecular tools enabling target engagement studies in a live cell environment with intact cell membranes, are of special relevance for identifying the most promising drug candidates for further development. Therefore, in this manuscript, we review recent approaches that leverage cellular target engagement studies for the IABS and thus play a critical role in the evaluation of IABS‐targeted GPCR ligands as potential therapeutic agents.

## Small Molecule Tools Targeting the IABS

Radioligands are among the most commonly used molecular tools for studying drug–target binding interactions in the field of GPCR research. One reason for their wide application is that radioligand binding assays allow measurements with native target proteins. This has the advantage that no modifications of the target protein, which eventually affect its druggability, subcellular distribution or expression level, are required. In addition, to transform a small molecule ligand into a radioactive tracer, usually only minor structural modifications are sufficient, such as the replacement of a hydrogen atom (^1^H) by radioactive tritium (^3^H, beta emitter, t_1/2_=12.3 years). Thus, radioligands have very similar binding affinities and selectivity profiles compared to their parent non‐radioactive ligands. Further, the readout of radioligand binding assays via scintillation counting, which is most frequently used for this purpose, is both highly robust and sensitive.^[14]^ Given these advantages, it is not surprising that radioligands have also played a pivotal role in the discovery of the IABS. Several tritium‐labelled intracellular allosteric antagonists, including the CCR1/CCR2‐targeted [^3^H]‐CCR2‐RA‐[*R*],^[15]^ the CCR9‐targeted [^3^H]‐vercirnon, and the CXCR1/2‐targeted [^3^H]‐Sch527123^[16]^ (also referred to as [^3^H]‐navarixin) were successfully applied in radioligand binding assays to assess drug interactions with the IABS of the respective GPCR. However, in the case of intracellular target binding sites, such as the IABS, the application of radioligand binding assays is mainly limited to cell‐free conditions and not well‐suited for cell‐based studies. The reason for that is, that radioligand binding assays mostly rely on a heterogeneous assay protocol, which means that washing steps are required to remove the unbound radioligand prior to assay readout via scintillation counting. Whereas the removal of the extracellular unbound ligand fraction can easily be accomplished by buffer exchange, the separation of intracellular unbound ligand fraction is not straightforward, due to the membrane barrier. More general drawbacks of radioligand binding assays are high infrastructure requirements according to radiation protection measures and the production of radioactive waste. In order to provide new approaches enabling non‐isotopic and cell‐based binding studies for the IABS of GPCRs, we and others have recently developed new small molecule‐based tools that are discussed below.

**Figure 3:**
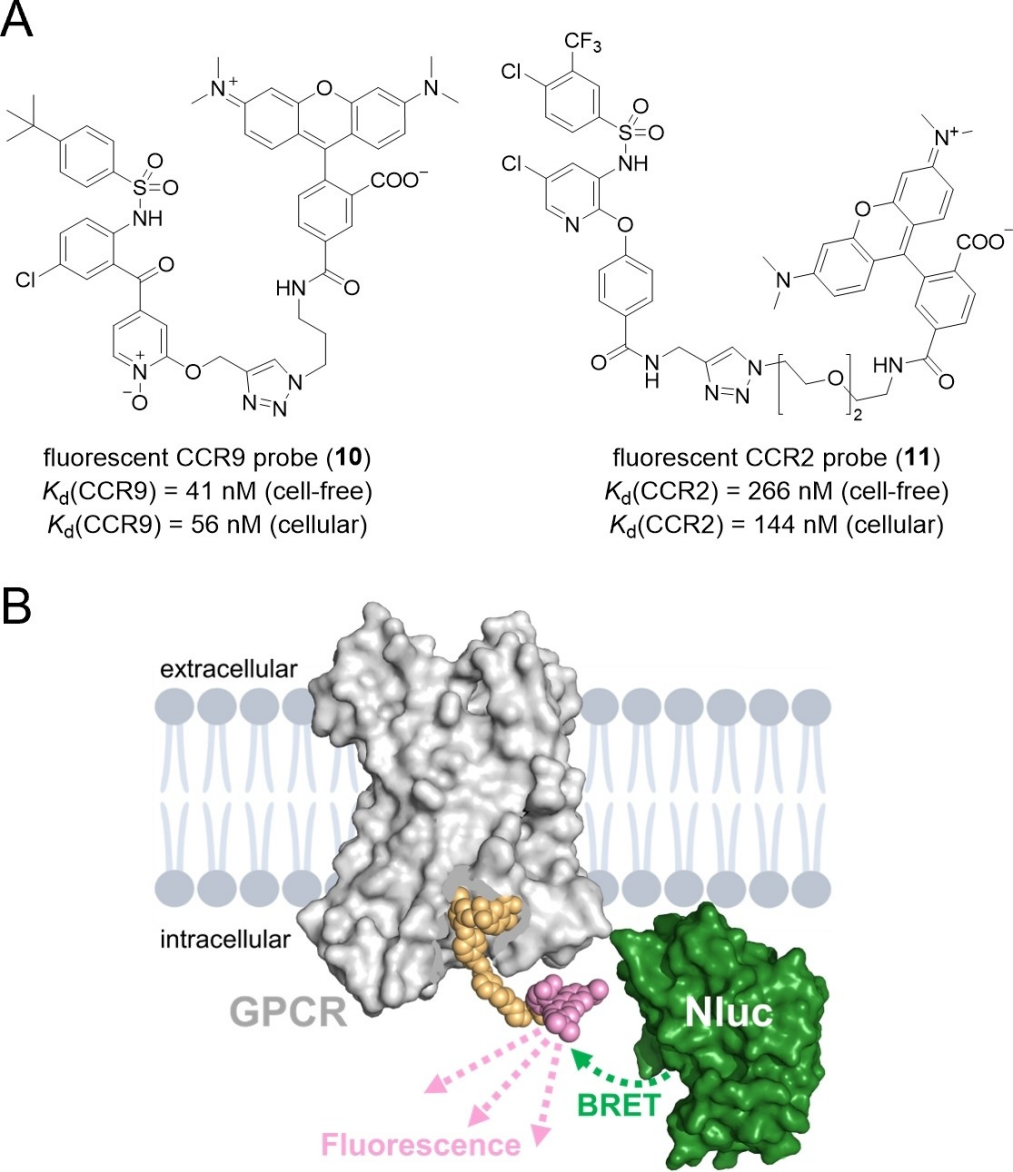
Fluorescent ligands targeting the intracellular allosteric binding site (IABS) of GPCRs. A) Chemical structures and affinity data for the fluorescent ligands **10** and **11** targeting the IABS of CCR9 and CCR2, respectively.[^13^, ^19^] B) Schematic representation of a NanoBRET strategy to detect binding to the IABS of GPCRs.

Similar to radioligands, fluorescent ligands have had a major impact on drug discovery campaigns in the field of GPCR research over the past years.^[17]^ Although the synthesis of small molecule‐based fluorescent ligands is far from trivial, as major modifications at the parent ligand are required, i. e. attachment of a relatively bulky fluorophore, it requires lower level regulatory and safety precautions compared to the preparation of radioligands. Further, fluorescent ligands are highly versatile tools for studying GPCR‐ligand‐interactions and can be used for a range of different applications, including fluorescence microscopy, fluorescence polarization (FP), fluorescence resonance energy transfer (FRET), and bioluminescence resonance energy transfer (BRET). The latter three techniques (FP, FRET, BRET) do not require a removal of the unbound fluorescent tracer molecule prior to assay readout, and thus can be used in a homogeneous, fast “mix‐and‐measure” setup.^[17]^ This is a major advantage compared to radioligand binding assays, and especially relevant for detecting ligand binding to intracellular target sites in a live cell environment. However, up until recently, no fluorescently labeled small molecule ligands had been developed that would allow a direct assessment of ligand binding to the IABS of GPCRs. In 2021, our group reported the first IABS‐targeted fluorescent probe for the C−C chemokine receptor type 9 (CCR9).^[13]^ The design of our fluorescent CCR9 probes, exemplified by **10** (Figure 3A), was based on the highly potent and selective intracellular CCR9 antagonist vercirnon (**5**). A conjugation of the cell‐permeable tetramethylrhodamine (TAMRA) fluorophore to the vercirnon‐based pharmacophore was enabled by a final stage Cu(I)‐catalyzed Huisgen cycloaddition.^[18]^ To evaluate receptor binding of the IABS‐targeted fluorescent probes, a NanoBRET‐based binding assay was established (Figure 3B). Therefore, we labeled CCR9 at its intracellular C‐terminus with a small and bright luciferase variant (nanoluciferase, Nluc).^[20]^ This NanoBRET assay setup enabled thermodynamic and kinetic binding studies. In a cell‐free NanoBRET‐based setup, **10** allowed the identification and characterization of drug‐like as well as low molecular weight CCR9 ligands in a highly accurate and straightforward manner. Kinetic binding studies with **10** revealed that the interaction between CCR9 and the vercirnon scaffold is characterized by a long residence time of 90 min, which rationalizes the high affinity and outstanding CCR9 selectivity of vercirnon (**5**). Most importantly, by applying **10** in a cellular NanoBRET assay, we were able to detect and accurately quantify target engagement for the IABS of CCR9 in a live cell environment. Intracellular CCR9 binding of **10** and its displacement by non‐fluorescent competitors, such as vercirnon (**5**), was also monitored by fluorescence microscopy. Thus, **10** represents an unprecedented tool that enables the quantification as well as visualization of ligand binding to the IABS of CCR9 in a cellular environment. In addition, the application of **10** as a screening tool in cell‐free and cellular NanoBRET assays enabled the discovery of the 4‐aminopyrimidine AAA30 (**9**) as a new intracellular CCR9 antagonist with improved affinity and antagonistic activity compared to the clinical candidate vercirnon (**5**).^[13]^

In a similar approach, we very recently developed **11** (Figure 3A) as a fluorescent ligand targeting the IABS of the chemokine receptor CCR2.^[19]^ As starting points for the design of our fluorescent CCR2 tracers, we considered the intracellular allosteric CCR2 antagonist CCR2‐RA‐[*R*] (**3**, Figure 1) as well as biarylsulfonamide‐based pharmacophores that were previously reported to target the IABS of CCR2.[^3^, ^15^, ^21^] Among the synthesized fluorescent CCR2 ligands, **11** showed the highest CCR2 affinities (Figure 3A). The CCR2 binding unit of **11** is based on a biarylsulfonamide pharmacophore previously reported by Wang et al.^[21c]^ In analogy to our fluorescent CCR9 ligands, **11** also enabled thermodynamic as well as kinetic NanoBRET‐based binding assays, fragment screening, and cellular target engagement studies.

**Figure 4:**
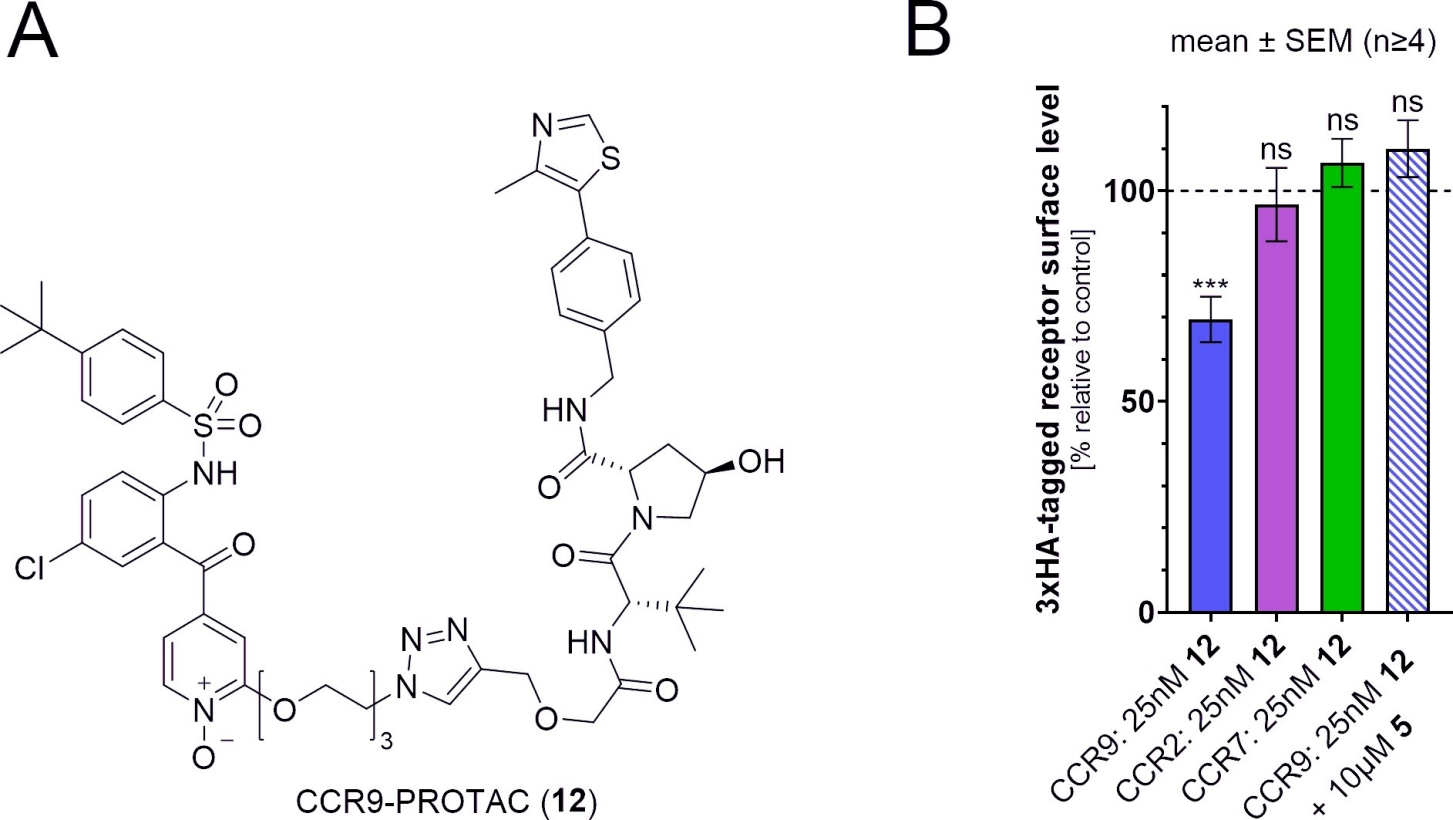
PROTAC targeting the intracellular allosteric binding site (IABS) of CCR9. A) Chemical structure of the CCR9‐PROTAC (**12**). B) ELISA results indicate selective degradation of CCR9 and that PROTAC‐induced CCR9 degradation can be counteracted by the intracellular allosteric antagonist vercirnon (**5**).^[13]^

Over the last years, the approach of targeted protein degradation (TPD), induced by means of so‐called proteolysis‐targeting chimeras (PROTACs), has gained much attention, due to several key advantages compared to standard inhibition of protein function by small molecules. The two most relevant advantages of PROTACs are their catalytic mode of action and a durable inhibition of protein function as a consequence of irreversible target protein degradation.^[22]^ Besides these pharmacological benefits, which have implications for basic research and clinical applications, PROTACs can also be utilized as molecular tools for studying cellular target engagement. By displacing the PROTAC from its intracellular binding site, an unlabeled small molecule competitor can prevent PROTAC‐induced target protein degradation, which can be assessed via immunosorbent assays like Western blot or enzyme‐linked immunosorbent assay (ELISA).[^13^, ^23^] This experiment is commonly performed in the course of PROTAC validation, but it can just as well be used as a method for cellular target engagement. While the PROTAC technology had a significant impact on drug discovery in general, its application to degrade GPCRs is still in its beginnings. To our best knowledge, only two GPCR‐targeted PROTACs for the α_1A_‐adrenergic receptor (α_1A_‐AR) and CCR9, respectively, have been reported thus far.[^13^, ^24^] With our Von Hippel‐Lindau protein (VHL)‐mediated and vercirnon‐based CCR9‐PROTAC (**12**), we developed the first GPCR‐targeted PROTAC that utilizes the IABS as a drug target site.^[13]^ With a maximum degradation effect of 31 % at a concentration of 25 nM, **12** evoked a limited but statistically significant and selective degradation of CCR9 (Figure 4B). Further, the degradation effect of **12** can be counteracted by competition with vercirnon (**5**), thereby demonstrating that PROTACs are indeed suitable tools to study cellular target engagement for the IABS of GPCRs.^[13]^ Current studies in our lab are aiming at increasing the degradation efficiencies of our CCR9 degraders, which will also increase their robustness as molecular tools to assess cellular target engagement. Like NanoBRET‐based approaches for cellular target engagement, the PROTAC‐based protocol relies on the availability of a functionalized ligand but does not necessarily require specific modifications of the targeted protein. It should also be noted, that for ligands showing a reduction of PROTAC‐mediated protein degradation, an orthogonal target engagement assay should be performed, as an inhibition of the employed E3 ligase or the proteasome might lead to false positive results.

The covalent CCR2 antagonist **7** (see Figure 2), recently reported by Ortiz Zacarías et al., opens new opportunities for further alternative approaches to detect cellular target engagement for the IABS. By targeting Cys75, a cysteine residue that is located at the entrance of the IABS and unique to CCR2 among all chemokine receptors, **7** is highly selective toward CCR2.^[11]^ As recent methods in protein mass spectrometry (MS) allow the detection of covalent small‐molecule‐protein adducts in a cellular background,^[25]^ **7** lays the foundation for future protein MS‐based studies to investigate cellular target engagement for the IABS.

## Summary and Outlook

Cellular target engagement studies are of fundamental importance in order to confirm relevant drug targets and to evaluate the cellular on‐target interactions of biologically active compounds. For the IABS of GPCRs, which is a promising target site for the development of novel drugs, substantial progress in establishing small molecule tools to study cellular target engagement has been made in recent years. With the development of IABS‐targeted fluorescent ligands and their application in NanoBRET‐based binding assays, a new approach has been established that enables an accurate and straightforward assessment of cellular target engagement for intracellular allosteric antagonists, thereby overcoming the limited applicability of radioligands for this purpose. Future approaches to study cellular target engagement for the IABS might benefit from recently reported IABS‐targeted tools, i. e. PROTACs and covalent ligands. Taking into account that tool compounds such as IABS‐targeted fluorescent ligands or PROTACs are not exactly drug‐like, due to their high molecular weight, membrane permeability should be considered as an important parameter during their development. Thus, the design of these heterobifunctional ligands should be based on intracellular GPCR‐ligands and fluorophores (e. g. TAMRA,^[26]^ for fluorescent tracers) or E3 ligands (e. g. VHL ligands,^[27]^ for PROTACs) with a sufficient membrane permeability. For the IABS‐targeted fluorescent ligands or PROTACs, suitable membrane permeability was indirectly shown by their successful application in cell‐based assays.[^13^, ^19^] For these IABS‐targeted tools, the mechanism of membrane permeation has not been elucidated so far. However, as other PROTACs as well as TAMRA‐labelled fluorescent ligands for intracellular target proteins were reported to pass the cell membrane mainly via passive diffusion,[^26b^, ^27^, ^28^] a similar mechanism can also be assumed for IABS‐targeted PROTACs and fluorescent tracers. In addition to the small molecule tools presented in this review, also fluorescently labeled peptides and single domain antibodies (nanobodies) have been developed to study binding to the IABS of GPCRs.^[29]^ A systematic application of different orthogonal target engagement methods will further aid the development of high‐quality probes and drug candidates for the IABS of GPCRs. The importance of molecular tools to study target engagement for the IABS is further underlined by recent reports indicating that a druggable IABS is a much more general feature of GPCRs than previously assumed and not only a phenomenon limited to β_2_AR and chemokine receptors.^[8]^

## Conflict of interest

The authors declare no conflict of interest.

1

## Biographical Information

*Max E. Huber studied pharmacy at the Friedrich‐Alexander‐Universität Erlangen‐Nürnberg (FAU) and received his license as a pharmacist in 2020. Currently, he is a PhD student in the Schiedel group at the FAU. His PhD project is focused on the development of small molecule ligands and molecular tools targeting the intracellular allosteric binding site of the chemokine receptor CCR9*.

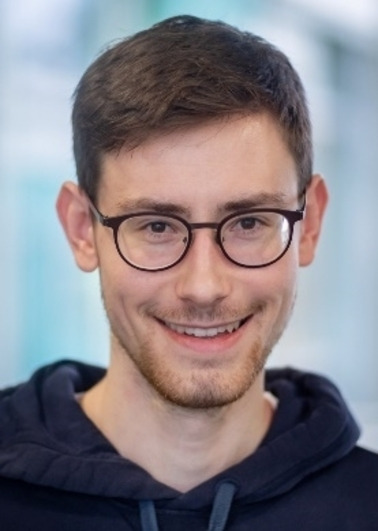

## Biographical Information

*Lara Toy studied Molecular Science (MSc) at the FAU. Currently, she is a PhD student in the Schiedel group at the FAU. Her PhD project is focused on the development of small molecule ligands and molecular tools targeting the intracellular allosteric binding site of the chemokine receptor CCR2*.

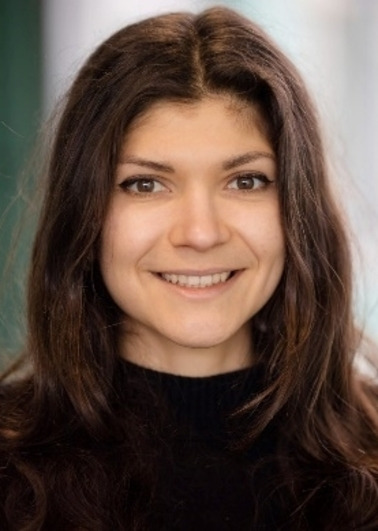

## Biographical Information

*Maximilian F. Schmidt studied pharmacy at the FAU and received his license as a pharmacist in 2017. During his doctoral studies under the supervision of Peter Gmeiner and Tim Clark, his work was focussed on molecular modelling and molecular dynamics simulations of GPCR‐ligand complexes. Currently, he is working in the hospital pharmacy at the University Hospital Erlangen*.

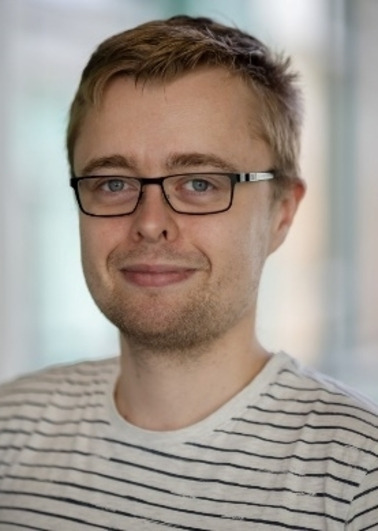

## Biographical Information

*Dorothee Weikert is a junior group leader at the Department of Chemistry and Pharmacy of the FAU. During her PhD at the FAU under the supervision of Kristina Friedland and Peter Gmeiner, she joined the lab of Michael Bouvier at the University of Montreal for a research stay. A second research stay, in the lab of Brian Kobilka at the Stanford University, followed her PhD. In 2016, she returned to the FAU and is working with her group on BRET‐based studies of GPCR activation and protein‐protein interactions, like receptor dimerization, especially in the context of bivalent ligands*.

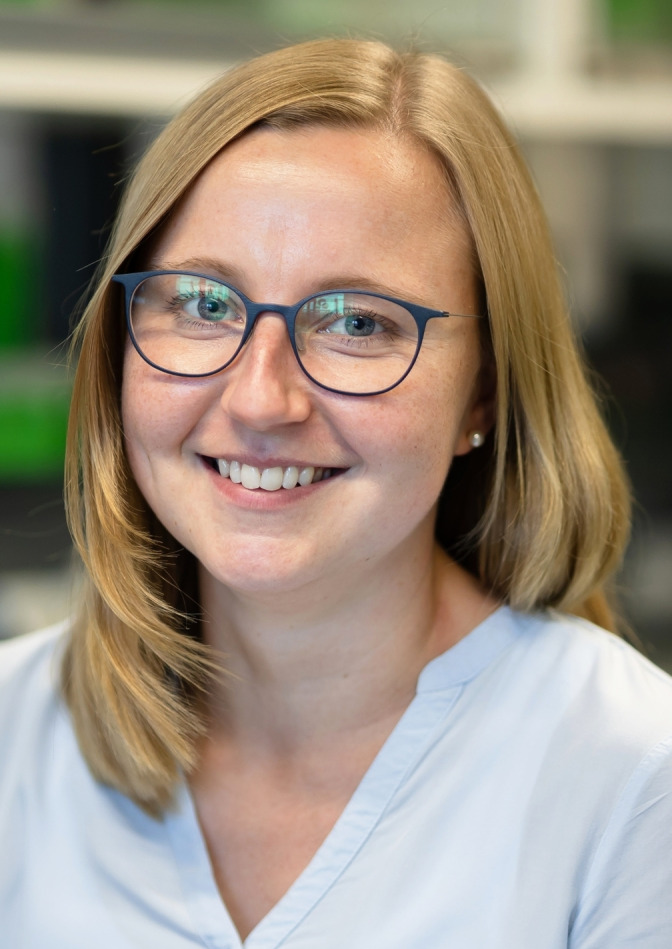

## Biographical Information

*Matthias Schiedel is a junior group leader (Liebig Fellow) at the Department of Chemistry and Pharmacy of the FAU Erlangen‐Nürnberg. After working in the field of epigenetic drug discovery for his PhD at the University of Freiburg with Manfred Jung and his postdoc at the University of Oxford with Stuart Conway, he joined the field of GPCR research and is now working with his group on novel approaches for targeting the intracellular allosteric binding site of GPCRs*.

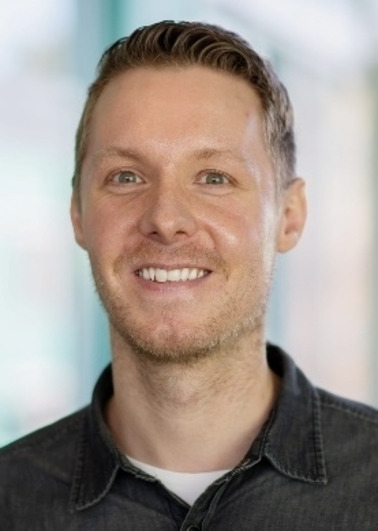

## Data Availability

Data sharing is not applicable to this article as no new data were created or analyzed in this study.
